# Clinical Factors Associated With Early Ketamine Initiation Among Mechanically Ventilated Adults: A Retrospective MIMIC‐IV Cohort Study

**DOI:** 10.1155/anrp/3312071

**Published:** 2026-11-04

**Authors:** Mary Ivasyutyn, Gaurav Prabhu, Saajan Bhakta

**Affiliations:** ^1^ Department of Research Kansas Health Science University-Kansas College of Osteopathic Medicine (KansasCOM) Wichita Kansas USA; ^2^ Medical Intensive Care Unit Riverside Regional Medical Center Newport News Virginia USA

**Keywords:** intensive care units, invasive mechanical ventilation, ketamine, sedation

## Abstract

**Introduction:**

Ketamine is used for analgesia and sedation in mechanically ventilated ICU patients; however, the clinical factors associated with its early initiation remain poorly characterised.

**Methods:**

We conducted a retrospective cohort study using the Medical Information Mart for Intensive Care IV Version 3.1 among adults receiving invasive mechanical ventilation and continuous first‐line sedation with propofol, dexmedetomidine or midazolam. Early ketamine was defined as recorded administration by a continuous infusion or medication push within 48 h after qualifying first‐line sedation began. Concurrent ventilation or continued first‐line sedation was not required. Associations were assessed using multivariable logistic regression with patient‐clustered robust standard errors.

**Results:**

Among 24,363 qualifying ICU stays in 22,396 patients, 226 stays (0.9%) met the early‐ketamine definition. The primary complete‐case analysis included 21,342 stays and 196 early‐ketamine events. Surgical/trauma intensive care unit admission (adjusted odds ratio 2.05, 95% confidence interval 1.39–3.04; *p* < 0.001), Black race (adjusted odds ratio 1.63, 95% confidence interval 1.05–2.53; *p* = 0.028) and Stage 3 acute kidney injury (adjusted odds ratio 1.67, 95% confidence interval 1.10–2.53; *p* = 0.016) were associated with higher odds of early ketamine initiation. Cardiac ICU admission and higher P/F ratio were associated with lower odds. Mean PEEP showed a nonlinear association (*p* = 0.005). Of 226 starts, 165 (73.0%) occurred during recorded invasive ventilation. Estimates were similar after bolus exclusion, whereas restricting initiation to recorded ventilation attenuated several associations, including surgical/trauma ICU admission (adjusted odds ratio 1.49; 95% CI, 0.97–2.31).

**Discussion:**

Early ketamine administration was uncommon under the study definition and did not always begin during recorded invasive ventilation. Associations were similar after bolus exclusion, but some attenuated when initiation was restricted to recorded ventilation. These findings describe patterns of ketamine use and do not establish treatment indications or therapeutic benefits.

## 1. Introduction

Patients receiving invasive mechanical ventilation may require analgesia and sedation for pain, anxiety and ventilator dyssynchrony, as well as to support ongoing ICU care [1, 2]. Contemporary ICU care generally favours light, goal‐directed sedation when clinically appropriate, as deeper sedation has been associated with prolonged mechanical ventilation, longer ICU stay and delirium [1, 3, 4]. The use of benzodiazepine‐based sedation has decreased because of its association with delirium and prolonged sedation, while nonbenzodiazepine sedatives such as propofol and dexmedetomidine are generally preferred [1, 2]. However, achieving adequate sedation without complications such as hypotension, bradycardia, delirium or excessive opioid exposure remains challenging, particularly in patients with agitation or severe respiratory illness [5, 6]. Therefore, ketamine has attracted increasing interest as a sedative and analgesic agent because of its distinct pharmacological properties.

Ketamine is an N‐methyl‐D‐aspartate (NMDA) receptor antagonist that provides both sedative and analgesic effects and may also produce bronchodilation and cardiovascular stimulation, characteristics that may be advantageous in selected critically ill patients [3, 7–9]. However, ketamine is also associated with adverse effects, including hypertension, tachycardia, agitation and hallucinations [5, 8, 10]. Evidence supporting routine ketamine use for ICU sedation remains limited. The 2018 PADIS guideline suggested low‐dose ketamine as an adjunct to opioids in postsurgical ICU patients, although this recommendation was based on limited evidence [1]. More recent literature has largely focused on outcomes such as opioid utilisation, sedation depth, delirium, ICU length of stay and duration of mechanical ventilation [5, 9, 11]. Although some evidence suggests that ketamine may reduce opioid requirements in critically ill patients, consistent improvements in duration of mechanical ventilation, ICU length of stay or mortality have not been demonstrated [9, 11]. Considerable variation also exists across studies in ketamine dosing, patient populations and indications for use [9, 11]. Far less is known about how often ketamine is administered soon after conventional continuous sedation begins and which patient and ICU characteristics are associated with its initiation [5, 12, 13].

We, therefore, examined the frequency of early ketamine administration within 48 h after the first qualifying continuous sedative infusion among ICU stays involving invasive mechanical ventilation. We also assessed patient characteristics, respiratory support measures and ICU setting associated with this early initiation. The exposure window was anchored to sedation initiation, not the entire period of mechanical ventilation, and could include ketamine started outside a recorded ventilation interval. Our analysis concerns treatment selection and does not establish the clinical indication for ketamine or its effect on patient outcomes.

## 2. Methods

We conducted a retrospective observational cohort study using the Medical Information Mart for Intensive Care IV (MIMIC‐IV), Version 3.1, a publicly available, deidentified electronic health record database containing hospital and intensive care data from Beth Israel Deaconess Medical Center, Boston, MA, USA [14, 15]. Access to MIMIC‐IV was obtained through PhysioNet after completion of the required credentialing and data‐use agreement [14, 16]. The study was reported in accordance with the Strengthening the Reporting of Observational Studies in Epidemiology (STROBE) statement [17]. The database contains deidentified data collected under institutional approval; therefore, no additional patient consent or institutional ethical approval was required for this secondary analysis.

We identified ICU stays among adults aged ≥ 18 years who received invasive mechanical ventilation and a qualifying continuous first‐line sedative infusion. First‐line sedation was defined as the first qualifying continuous infusion of propofol, dexmedetomidine or midazolam. The qualifying ICU stay was the primary unit of analysis. The final cohort comprised 24,363 qualifying ICU stays among 22,396 unique patients; therefore, some patients contributed more than one qualifying stay. Repeated stays were retained because each represented a distinct episode of mechanical ventilation and sedation management and were accounted for statistically using patient‐level clustering.

For each qualifying ICU stay, invasive mechanical ventilation and first‐line continuous sedation were identified from time‐stamped MIMIC‐IV records. The primary outcome was early ketamine initiation, defined as a recorded ketamine administration, either continuous infusion or medication push beginning 0–48 h after the first qualifying continuous infusion of propofol, dexmedetomidine or midazolam. Ongoing first‐line sedation and concurrent active ventilation were not required at ketamine initiation. Administration before first‐line sedation or after the 48‐h window was not counted as early. This yielded 226 early‐ketamine stays, including 220 with identifiable continuous‐infusion records and six with medication‐push records only. The dosing analysis was restricted to the 220 infusion stays, and both administration types were included in the primary exposure definition. To describe recorded ventilation timing, the ketamine start timestamp was compared with all recorded invasive‐ventilation intervals within the same ICU stay and classified as during an interval, after the last interval, before the first interval or between intervals. Initiation was considered during an interval when it occurred at or after its start and before its end. A separate timing analysis excluded stays in which the qualifying sedative ended before ventilation or began more than 12 h after ventilation initiation. This addressed sedative–ventilation alignment rather than restricting ketamine initiation to active ventilation.

Candidate covariates were selected a priori based on clinical relevance and MIMIC‐IV availability and included age, sex, race/ethnicity, body weight, first‐line sedative, Charlson Comorbidity Index, Sequential Organ Failure Assessment (SOFA) score, ICU type, PaO_2_/FiO_2_ (P/F) ratio, mean positive end‐expiratory pressure (PEEP), mean arterial pressure (MAP), vasopressor burden, Stage 3 acute kidney injury (AKI) and Sepsis‐3 status. Stage 3 AKI used first ICU Day KDIGO staging. Variable‐specific measurement windows and aggregation rules are provided in Supporting Table S4. In particular, mean PEEP and vasopressor burden included entire‐stay measurements that could extend beyond ketamine initiation. Because measurement periods were not uniform across covariates, these variables were interpreted as characteristics associated with treatment initiation rather than strictly pre‐exposure predictors.

### 2.1. Data Extraction

MIMIC‐IV v3.1 data were accessed through PhysioNet and implemented locally in a PostgreSQL database. Data extraction and cohort construction were performed using Navicat (Version 17.0) as the graphical interface for PostgreSQL. Where available, validated MIMIC‐IV‐derived concepts and official MIT‐LCP SQL scripts from the MIMIC Code Repository on GitHub were used to generate standardised variables, including severity scores, comorbidity measures, vital signs, ventilator parameters and other derived clinical features. Study‐specific variables not available in the official concept library, including first‐line continuous sedative exposure, mechanical ventilation/sedation timing, ketamine administration timing and early ketamine status, were derived using custom PostgreSQL SQL queries from time‐stamped MIMIC‐IV event data.

### 2.2. Ketamine Dosing Characterisation

For the additional descriptive analysis, ketamine records were identified in MIMIC‐IV inputevents using ketamine itemid 221712 with both ordercategoryname = ‘01‐Drips’ and ordercategorydescription = ‘Continuous Med’ as separate required fields. For the 220 stays with continuous‐infusion records, the observational window extended from ketamine_start through ketamine_end and was not truncated at 48 h. Qualifying later infusion segments were retained. Recorded segment rates were standardised to mg/kg/h. Initial infusion rate was taken from the earliest qualifying segment. A stay‐level time‐weighted mean rate was calculated by summing each recorded segment rate multiplied by that segment’s own duration and dividing by the total contributing segment duration. For cumulative infusion duration, overlapping or contiguous segments were merged, and gaps were excluded. Medication pushes were excluded from infusion dose characterisation.

### 2.3. Statistical Analysis

Age and weight were summarised as mean (SD); other continuous variables were summarised as median (IQR) and categorical measures as counts and percentages. Group comparisons used Wilcoxon rank‐sum, Pearson’s chi‐squared or Fisher’s exact tests as appropriate. Complete‐case multivariable logistic regression provided adjusted odds ratios and 95% confidence intervals for early ketamine initiation. Patient‐clustered robust standard errors accounted for repeat ICU stays. All eligible stays were considered without a priori power calculation. Statistical significance was defined as *p* < 0.05.

Mean PEEP was modelled using a natural cubic spline with three degrees of freedom. Overall PEEP association and departure from linearity were assessed using patient‐clustered robust Wald tests. P/F ratio was retained as a linear term because a spline‐based assessment showed no evidence of nonlinearity. Two post hoc sensitivity analyses separately excluded early‐ketamine stays with bolus‐only administration and early‐ketamine stays in which initiation occurred outside recorded invasive‐ventilation intervals. Each analysis retained the original no‐early‐ketamine comparison stays and used the primary complete‐case sample, covariates, PEEP spline basis and patient‐clustered robust standard errors. Other sensitivity analyses included the exclusion of the P/F ratio from the model, multiple imputation by chained equations, Firth‐penalised logistic regression, a stricter mechanical ventilation/sedation timing definition and restriction to the first qualifying ICU stay per patient.

Multiple imputation by chained equations was performed as a sensitivity analysis, with estimates pooled using Rubin’s rules (Supporting Table S3). Apparent and optimism‐corrected AUC, Brier score and calibration were assessed, with internal validation based on 1000 patient‐level bootstrap resamples. Additional imputation details and model performance are provided in Supporting Tables S3 and S2. Statistical analyses and model validation were performed in *R* Version 4.6.1.

## 3. Results

The cohort included 24,363 qualifying ICU stays among 22,396 patients. Recorded early ketamine administration occurred in 226 qualifying ICU stays (0.9%), whereas 24,137 stays did not meet this definition. The early‐ketamine group included 220 stays with identifiable continuous‐infusion records and six with medication‐push records only. The flowchart of this study is depicted in Figure 1. Patients in early ketamine stays were younger on average and had a lower P/F ratio and higher mean PEEP than those in the comparison group. Surgical/trauma ICU stays were also more common in the early‐ketamine group. Additional cohort characteristics are presented in Table 1.

**FIGURE 1:**
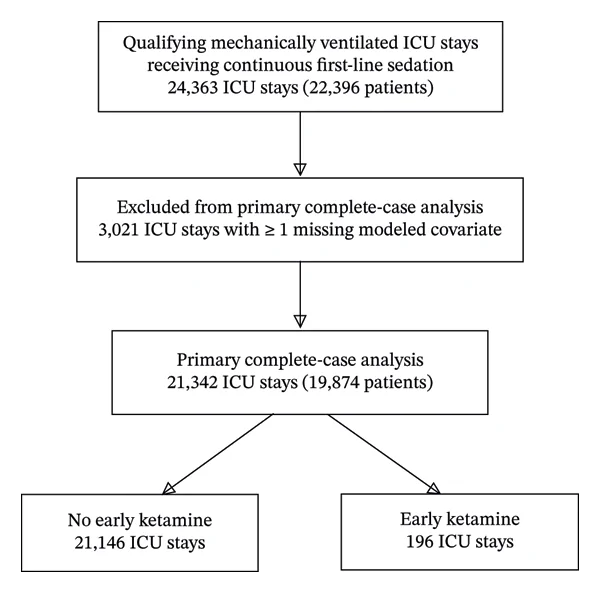
Study cohort and primary analytic sample.

**TABLE 1:** Clinical characteristics of qualifying mechanically ventilated ICU stays by early ketamine initiation.

Characteristic	Overall, *N* = 24,363	No early ketamine, *n* = 24,137	Early ketamine *n* = 226	*p* value
Age (years), mean (SD)	64.1 (15.8)	64.2 (15.8)	53.5 (16.0)	< 0.001
Sex				0.990
Female	9442 (38.8%)	9355 (38.8%)	87 (38.5%)	
Male	14,921 (61.2%)	14,782 (61.2%)	139 (61.5%)	
Race/ethnicity				0.026
White	15,752 (64.7%)	15,625 (64.7%)	127 (56.2%)	
Black	2068 (8.5%)	2036 (8.4%)	32 (14.2%)	
Asian	666 (2.7%)	661 (2.7%)	5 (2.2%)	
Hispanic	990 (4.1%)	978 (4.1%)	12 (5.3%)	
Other	938 (3.9%)	928 (3.8%)	10 (4.4%)	
Unknown/not reported	3949 (16.2%)	3909 (16.2%)	40 (17.7%)	
Weight (kg), mean (SD)	84.7 (24.4)	84.6 (24.3)	93.3 (30.4)	< 0.001
First‐line continuous sedative				0.513
Dexmedetomidine	345 (1.4%)	340 (1.4%)	5 (2.2%)	
Midazolam	4851 (19.9%)	4807 (19.9%)	44 (19.5%)	
Propofol	19,167 (78.7%)	18,990 (78.7%)	177 (78.3%)	
Charlson Comorbidity Index	4.0 (3.0, 7.0)	4.0 (3.0, 7.0)	3.0 (1.0, 5.0)	< 0.001
APS III	42.0 (30.0, 60.0)	42.0 (30.0, 60.0)	47.0 (32.0, 65.0)	0.022
SOFA score	5.0 (3.0, 8.0)	5.0 (3.0, 8.0)	5.0 (3.0, 9.0)	0.597
OASIS	36.0 (31.0, 41.0)	36.0 (31.0, 41.0)	37.0 (31.0, 42.0)	0.067
Minimum Glasgow Coma Scale (GCS) score	15.0 (14.0, 15.0)	15.0 (14.0, 15.0)	15.0 (14.0, 15.0)	0.005
ICU type				< 0.001
Medical ICU	4831 (19.8%)	4778 (19.8%)	53 (23.5%)	
Surgical/trauma ICU	6534 (26.8%)	6414 (26.6%)	120 (53.1%)	
Cardiac ICU	9483 (38.9%)	9465 (39.2%)	18 (8.0%)	
Neuro ICU	999 (4.1%)	993 (4.1%)	6 (2.7%)	
Mixed/Other ICU	2516 (10.3%)	2487 (10.3%)	29 (12.8%)	
PaO_2_/FiO_2_ ratio	175.0 (100.0, 271.0)	175.4 (101.0, 271.9)	124.5 (77.8, 200.0)	< 0.001
Mean FiO_2_ (%)	50.0 (43.0, 57.9)	50.0 (43.0, 57.9)	50.0 (43.9, 55.8)	0.681
Mean PEEP (cm H_2_O)	5.1 (5.0, 6.8)	5.1 (5.0, 6.8)	7.6 (5.0, 10.3)	< 0.001
Mean arterial pressure (mmHg)	75.8 (70.8, 82.1)	75.8 (70.8, 82.0)	79.1 (72.8, 86.2)	< 0.001
Vasopressor use	15,768 (64.7%)	15,613 (64.7%)	155 (68.6%)	0.250
Number of vasopressors				0.130
0	8628 (35.4%)	8557 (35.5%)	71 (31.4%)	
1	9271 (38.1%)	9189 (38.1%)	82 (36.3%)	
≥ 2	6464 (26.5%)	6391 (26.5%)	73 (32.3%)	
Maximum lactate (mmol/L)	2.4 (1.6, 3.8)	2.4 (1.6, 3.8)	1.8 (1.2, 2.9)	< 0.001
Maximum creatinine (mg/dL)	1.1 (0.8, 1.6)	1.1 (0.8, 1.6)	1.1 (0.8, 1.9)	0.245
KDIGO acute kidney injury stage, first ICU day				0.020
No AKI	7465 (30.6%)	7408 (30.7%)	57 (25.2%)	
Stage 1	4865 (20.0%)	4823 (20.0%)	42 (18.6%)	
Stage 2	9384 (38.5%)	9295 (38.5%)	89 (39.4%)	
Stage 3	2648 (10.9%)	2610 (10.8%)	38 (16.8%)	
Renal replacement therapy, first ICU day	1274 (5.2%)	1253 (5.2%)	21 (9.3%)	0.009
Sepsis‐3 criteria met	17,081 (70.1%)	16,907 (70.0%)	174 (77.0%)	0.028

*Note:* Data are mean (SD), median (Q1, Q3) or *n* (%). Percentages are based on available observations; missing values were not imputed. Variable‐specific denominators may differ because of missing data. The cohort comprised 24,363 qualifying ICU stays among 22,396 unique patients; some patients contributed more than one qualifying ICU stay. Wilcoxon rank‐sum test for continuous variables; Pearson’s chi‐squared test for categorical variables; Fisher’s exact test for first‐line continuous sedative.

A separate continuous‐infusion dosing analysis included 220 of the 226 early‐ketamine ICU stays (97.3%) with identifiable continuous‐infusion records. Six stays had only drug‐push entries without identifiable continuous‐infusion records and were excluded from this descriptive analysis alone. Across the 220 stays, the median initial infusion rate was 0.103 mg/kg/h (IQR 0.100–0.300) and the median stay‐level time‐weighted mean rate was 0.341 mg/kg/h (IQR 0.192–0.603). Median cumulative recorded infusion duration was 46.74 h (IQR 18.60–148.45). Supporting Table S5 provides the complete descriptive dosing summary and ranges.

In the separate ventilation‐timing classification of all 226 early administrations, 165 (73.0%) began during recorded invasive ventilation, 28 (12.4%) after the last recorded interval, 23 (10.2%) before the first and 10 (4.4%) between intervals (Supporting Table S7). Among the 28 after the last interval, the median gap was 8.03 h (range 0.63–33.53), and 26 were more than one hour after the interval ended.

The complete‐case multivariable analysis included 21,342 qualifying ICU stays, 196 early‐ketamine events and 19,874 unique patients; 3021 stays had at least one missing model covariate. Surgical/trauma ICU admission, Black race compared with White race and Stage 3 AKI were associated with higher odds of early ketamine initiation. Cardiac ICU admission, higher P/F ratio, greater Charlson Comorbidity Index and higher SOFA score were associated with lower odds of early ketamine initiation. Mean PEEP showed a nonlinear association. Full primary regression estimates and *p* values are presented in Table 2, selected estimates are shown in the forest plot (Figure 2), and the nonlinear PEEP association appears in Supporting Figure S1.

**TABLE 2:** Multivariable factors associated with early ketamine initiation.

Variable	Adjusted OR (95% CI)	*p* value
Age, per 10‐year increase	0.93 (0.82–1.04)	0.208
Male vs. female	0.95 (0.71–1.29)	0.765
Black vs. White	1.63 (1.05–2.53)	0.028
Asian vs. White	1.15 (0.47–2.84)	0.761
Hispanic vs. White	1.05 (0.55–2.00)	0.878
Other vs. White	0.81 (0.39–1.69)	0.568
Unknown/not reported vs. White	0.74 (0.50–1.09)	0.126
Weight, per 10‐kg increase	0.99 (0.95–1.04)	0.795
Dexmedetomidine vs. propofol	0.82 (0.20–3.34)	0.782
Midazolam vs. propofol	0.73 (0.50–1.07)	0.110
Charlson Comorbidity Index	0.86 (0.79–0.93)	< 0.001
SOFA score, per 1‐point increase	0.94 (0.89–0.99)	0.017
Surgical/trauma ICU vs. medical ICU	2.05 (1.39–3.04)	< 0.001
Cardiac ICU vs. medical ICU	0.22 (0.11–0.42)	< 0.001
Neuro ICU vs. medical ICU	1.08 (0.41–2.86)	0.877
Mixed/Other ICU vs. medical ICU	1.10 (0.68–1.76)	0.704
P/F ratio, per 50‐unit increase	0.88 (0.82–0.96)	0.002
Mean PEEP, overall association	—	< 0.001
MAP, per 5‐mmHg increase	1.04 (0.97–1.12)	0.224
≥ 2 vasopressors vs. 0–1	1.07 (0.72–1.58)	0.745
Stage 3 AKI vs. Stage 0–2	1.67 (1.10–2.53)	0.016
Sepsis‐3: Yes vs. No	0.87 (0.61–1.24)	0.439

*Note:* Primary complete‐case multivariable logistic regression included 21,342 qualifying ICU stays among 19,874 unique patients, with 196 early‐ketamine events. Odds ratios are mutually adjusted for all covariates shown. Patient‐clustered robust standard errors accounted for repeated qualifying ICU stays within patients. Mean PEEP was modelled using a natural cubic spline with three degrees of freedom; therefore, a single adjusted odds ratio is not reported. Joint Wald tests using the patient‐clustered robust covariance matrix assessed the overall PEEP association (3 degrees of freedom; *p* < 0.001) and departure from linearity (2 degrees of freedom; *p* = 0.005). Reference categories were female sex, White race, propofol, Medical ICU, 0–1 vasopressors, AKI stages 0–2 and no Sepsis‐3.

**FIGURE 2:**
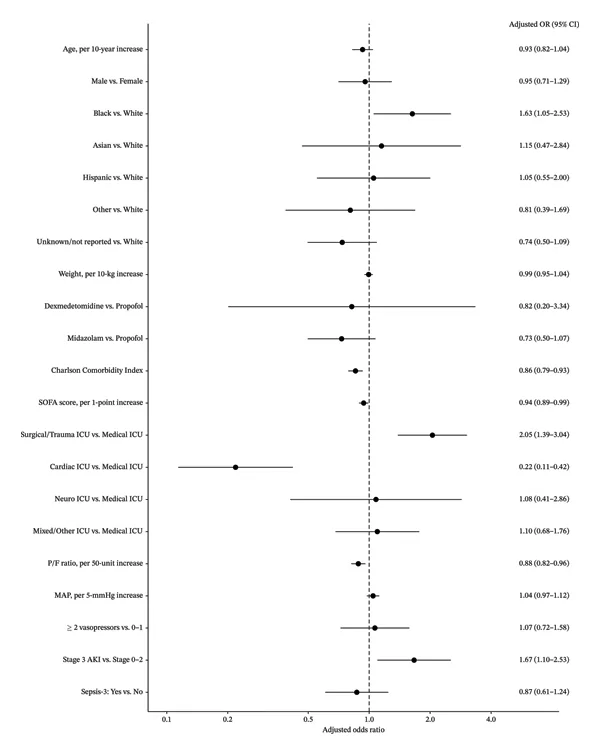
Adjusted associations with early ketamine initiation. Adjusted odds ratios and 95% confidence intervals are from the primary complete‐case multivariable logistic regression model with patient‐clustered robust standard errors. Mean PEEP is not displayed because it was modelled nonlinearly; its adjusted association is shown in Supporting Figure S1.

The principal associations were generally consistent across sensitivity analyses omitting P/F ratio, using multiple imputation and Firth‐penalised logistic regression, applying stricter initial ventilation and sedation timing rules or restricting the cohort to one ICU stay per patient (Supporting Table S1). The stricter initial timing analysis retained 23,443 stays and 220 early‐ketamine events before complete‐case restriction; these 220 events are distinct from the 220 infusion stays in the dosing subset. Missing‐data procedures are reported in Supporting Table S3.

In the post hoc bolus‐exclusion sensitivity analysis, removal of the four bolus‐only stays included in the primary complete‐case model left 21,338 stays among 19,871 patients, with 192 early‐ketamine events. Estimates were similar to the primary analysis (Supporting Table S6). A separate sensitivity analysis excluded the 53 early‐ketamine stays in the complete‐case sample whose initiation occurred outside recorded invasive ventilation, retaining the original comparison stays. This analysis included 21,289 stays among 19,825 patients, with 143 early‐ketamine events. Several associations attenuated, including surgical/trauma ICU admission (aOR 1.49, 95% CI 0.97–2.31), Black race compared with White race (aOR 1.46, 95% CI 0.87–2.45), SOFA score and P/F ratio. Both post hoc sensitivity analyses are presented in Supporting Table S6.

The apparent AUC of the primary model was 0.832 (95% CI 0.807–0.858), and the optimism‐corrected AUC was 0.815 after 1000 patient‐level bootstrap resamples. Additional model‐performance measures, including calibration and Brier scores, are presented in Supporting Table S2.

## 4. Discussion

Early ketamine initiation occurred in 0.9% of qualifying ICU stays. This frequency is specific to administration within 48 h of qualifying first‐line sedation and does not measure all ICU ketamine use. In adjusted analyses, surgical/trauma ICU admission, Black race compared with White race and Stage 3 AKI were associated with higher odds of early ketamine initiation. In contrast, cardiac ICU admission, higher P/F ratio, higher Charlson Comorbidity Index and higher SOFA score were associated with lower odds. Overall, the observed pattern suggests that ketamine initiation may reflect differences in ICU case mix, respiratory support requirements and sedation practice rather than overall illness severity alone. Bolus exclusion produced similar principal adjusted associations. However, restricting initiation to recorded invasive ventilation attenuated several associations, including surgical/trauma ICU admission, Black race compared with White race, SOFA score and P/F ratio (Supporting Table S6). Recorded timing showed that 165 administrations began during documented invasive ventilation, while 61 occurred before, between or after ventilation intervals. These timing categories cannot establish clinical reasons for treatment.

Previous studies of ketamine in mechanically ventilated ICU patients have primarily evaluated efficacy, safety, sedative requirements or clinical outcomes [9, 18–21]. Groth et al. reported substantial variation in ketamine use across critically ill populations [5], while a survey of Canadian intensivists also demonstrated variability in ketamine use and indications in the ICU [22]. A previous MIMIC‐IV study evaluated ketamine exposure in relation to mortality and length of stay among mechanically ventilated patients [23]. Our study instead describes the infrequent occurrence and associated characteristics of early ketamine administration after conventional sedation began. These are distinct questions, and their rates should not be compared directly without matching treatment definitions and observation windows.

The association with ICU type may reflect important differences in patient population and sedation requirements. Surgical and trauma patients often have substantial analgesic requirements in addition to sedation needs, including pain related to fractures, major operations, burns and repeated procedures. Ketamine has previously been used as an adjunct to analgesic and sedative therapy in trauma and surgical ICU populations [5, 24–27]. In contrast, the lower odds of ketamine initiation in cardiac ICUs may reflect differences in case mix and sedation culture rather than avoidance of ketamine itself. Patients in cardiac ICUs may have substantially different ventilation and sedation trajectories from patients with severe respiratory failure or polytrauma, and continuous ketamine has been reported as feasible in medical and cardiac ICU patients receiving mechanical ventilation [28]. Respiratory physiology was also associated with ketamine initiation. Mean PEEP showed a nonlinear association with early ketamine initiation, with adjusted odds generally increasing across higher PEEP values, whereas higher P/F ratio was associated with lower odds of initiation. These findings may identify patients requiring greater respiratory support or more complex ventilator management. In clinical practice, patients with greater respiratory support requirements may also be more difficult to keep comfortable or synchronous with mechanical ventilation and may require changes in ventilator management or deeper sedation. Ketamine has been evaluated during mechanical ventilation and in severe respiratory failure [19, 29]. However, these findings should not be interpreted as evidence that higher PEEP or lower oxygenation directly leads to ketamine use. Rather, these measures may reflect the clinical context in which additional analgesia or sedation is considered. Higher SOFA score and Charlson Comorbidity Index were associated with lower odds of ketamine initiation, whereas Stage 3 AKI was associated with higher odds. These findings suggest that global measures of illness severity and chronic comorbidity may not capture the specific bedside problems that prompt clinicians to add ketamine. Ketamine may be initiated in response to a particular sedation or analgesic problem rather than simply because a patient is more severely ill. Factors such as pain, agitation, ventilator dyssynchrony, increasing analgesic or sedative requirements and intolerance of conventional agents may be more relevant to this decision. Similarly, the number of vasopressors used in our analysis provides only a broad measure of haemodynamic support and does not capture changes in vasopressor dose over time. Previous studies have also demonstrated substantial variation in ketamine use and indications across ICU settings [5, 19, 22, 28, 30, 31].

Black race was associated with higher odds of early ketamine initiation compared with White race in the primary analysis. The reason for this finding cannot be determined from MIMIC‐IV, and race should not be interpreted as a biological determinant of ketamine use. The observed association could reflect differences in measured or unmeasured clinical characteristics, patterns of care or other institutional and structural factors. Further studies in other populations are needed to determine whether this association is reproducible.

### 4.1. Limitations

This study had several limitations. First, as a retrospective, single‐centre analysis, the study was subject to unmeasured confounding, practice variation and restricted generalisability. Secondly, the timing of several clinical variables was not uniform, and some measurements may have overlapped with or followed ketamine initiation. In particular, mean PEEP and vasopressor burden included entire stay measurements. Therefore, these findings describe associated clinical characteristics and do not establish temporal or causal effects. Thirdly, the sample size of the ketamine group was relatively small, given the large size of the MIMIC‐IV database. The primary complete‐case model included 196 early‐ketamine stays; restricting initiation to recorded invasive ventilation reduced this to 143 and attenuated several associations.

Fourthly, although MIMIC‐IV contains detailed longitudinal ICU data, the present analysis did not account for changes in sedative and opioid requirements, vasopressor dose or ventilator management over time, which may have influenced the decision to initiate ketamine. Although ketamine initiation was compared with all recorded invasive‐ventilation intervals, these records could not confirm exact clinical extubation timing or the indication for ketamine. Dosing characterisation was restricted to 220 stays with continuous‐infusion records, excluding six bolus‐only stays, and reflects recorded rather than independently verified delivered doses. Finally, MIMIC‐IV represents a single academic health system, which may limit the generalisability of the findings to other ICU settings.

## 5. Conclusion

In conclusion, early initiation of ketamine within 48 h of first‐line continuous sedation was uncommon (0.9% of qualifying ICU stays) and was associated with ICU type, respiratory physiology and selected clinical characteristics. Higher odds of initiation in surgical/trauma ICUs and associations with markers of greater respiratory support, together with lower odds in cardiac ICUs, suggest that ketamine initiation may reflect differences in case mix and sedation practice as well as patient‐level clinical factors. Associations were similar after excluding bolus‐only stays, but some attenuated when initiation was restricted to recorded invasive ventilation, highlighting the importance of treatment timing. These findings do not establish treatment indications or therapeutic benefits. Further prospective studies incorporating documented treatment indications and detailed trajectories of sedation, analgesia, ventilation and haemodynamics, together with validation in other settings, are needed to better define the bedside circumstances associated with ketamine use.

## Funding

This research received no specific grant from any funding agency in the public, commercial or not‐for‐profit sectors.

## Ethics Statement

MIMIC‐IV contains de‐identified health data collected under the institutional approvals governing the MIMIC database. Access to MIMIC‐IV was obtained through PhysioNet after completion of the required credentialing and data‐use agreement. This study was a secondary analysis of de‐identified MIMIC‐IV data. No separate KansasCOM Institutional Review Board exemption or determination was obtained for this analysis.

## Conflicts of Interest

The authors declare no conflicts of interest.

## Supporting Information

Additional supporting information can be found online in the Supporting Information section.

## Supporting information


**Supporting Information 1** Supporting Table S1. Sensitivity analyses of factors associated with early ketamine initiation. Supporting Table S2. Model performance, calibration and internal validation of the primary multivariable analysis. Supporting Table S3. Missing data and multiple‐imputation procedures. Supporting Table S4. Definitions and timing of variables included in the primary multivariable model. Supporting Table S5. Ketamine infusion dosing characteristics. Supporting Table S6. Primary and post hoc sensitivity analyses of early ketamine initiation. Supporting Table S7. Timing of early ketamine administration relative to recorded invasive ventilation. Supporting Figure S1. Adjusted nonlinear association between mean PEEP and early ketamine initiation. The solid line represents adjusted odds ratios estimated from the primary multivariable logistic regression model using a natural cubic spline with three degrees of freedom, with patient‐clustered robust standard errors; the shaded area represents the 95% confidence interval. Odds ratios are referenced to the median mean PEEP of 5.15 cm H₂O. Predictions are displayed over the 1st–99th percentile of observed mean PEEP values. Mean PEEP was associated with early ketamine initiation overall (*p* < 0.001), with evidence of nonlinearity (*p* = 0.005).

**Supporting Information 2** STROBE Statement—Completed Checklist for Cohort Studies

## Data Availability

MIMIC‐IV is available through PhysioNet to credentialed users who complete the required training and data‐use agreement. The data used for this study are subject to the access requirements of PhysioNet and cannot be redistributed directly by the authors.

## Appendix A Code Availability

Study‐specific SQL and R code used for cohort construction and statistical analysis will be made available by the corresponding author upon reasonable request.
