# Supplementary material for: Clinical Factors Associated With Early Ketamine Initiation Among Mechanically Ventilated Adults: A Retrospective MIMIC‐IV Cohort Study
Source: Anesthesiol Res Pract. 2026 Nov 4;2026:3312071. doi: 10.1155/anrp/3312071 (PMC13732811; doi:10.1155/anrp/3312071)
Supplement: Supplementary file 1 [file ANRP-2026-3312071-s001.docx]

**Supplementary Table S1.** Sensitivity analyses of factors associated with early ketamine initiation.

| **Variable** | **Primary analysis** | **No-P/F sensitivity** | **Multiple imputation** | **Firth regression** | **Strict-timing sensitivity** |
| --- | --- | --- | --- | --- | --- |
| Black vs White | 1.63 (1.05–2.53) | 1.55 (1.03–2.34) | 1.60 (1.06–2.42) | 1.63 (1.04–2.47) | 1.68 (1.08–2.60) |
| Charlson comorbidity index | 0.86 (0.79–0.93) | 0.87 (0.81–0.93) | 0.87 (0.81–0.94) | 0.85 (0.79–0.92) | 0.86 (0.79–0.93) |
| SOFA score, per 1-point increase | 0.94 (0.89–0.99) | 0.97 (0.92–1.01) | 0.94 (0.89–0.99) | 0.94 (0.89–0.99) | 0.95 (0.90–1.00) |
| Surgical/Trauma ICU vs Medical ICU | 2.05 (1.39–3.04) | 1.94 (1.35–2.77) | 2.15 (1.48–3.11) | 2.11 (1.44–3.11) | 2.21 (1.49–3.26) |
| Cardiac ICU vs Medical ICU | 0.22 (0.11–0.42) | 0.26 (0.14–0.45) | 0.27 (0.15–0.49) | 0.22 (0.12–0.40) | 0.23 (0.12–0.42) |
| P/F ratio, per 50-unit increase | 0.88 (0.82–0.96) | — | 0.88 (0.81–0.96) | 0.86 (0.79–0.93) | 0.86 (0.79–0.93) |
| MAP, per 5-mmHg increase | 1.04 (0.97–1.12) | 1.07 (1.00–1.14) | 1.06 (0.99–1.14) | 1.05 (0.97–1.13) | 1.05 (0.97–1.13) |
| ≥2 vasopressors vs 0–1 | 1.07 (0.72–1.58) | 1.21 (0.85–1.71) | 1.16 (0.81–1.66) | 1.11 (0.76–1.61) | 1.06 (0.73–1.56) |
| Stage 3 AKI vs Stage 0–2 | 1.67 (1.10–2.53) | 1.49 (1.00–2.22) | 1.59 (1.07–2.36) | 1.64 (1.06–2.47) | 1.53 (1.00–2.36) |
| Sepsis-3: Yes vs No | 0.87 (0.61–1.24) | 1.04 (0.74–1.46) | 0.90 (0.65–1.26) | 0.93 (0.65–1.35) | 0.99 (0.68–1.43) |

Values are adjusted odds ratios (95% confidence intervals). The primary analysis used patient-clustered robust standard errors. Mean PEEP was modelled nonlinearly using a natural cubic spline with three degrees of freedom in the primary model and is therefore not presented as a single odds ratio. Sensitivity analyses excluded the P/F ratio, used multiple imputation or Firth regression, or applied stricter mechanical ventilation/sedation timing criteria.

**Supplementary Table S1 (continued).** Sensitivity analysis restricted to the first qualifying ICU stay per patient.

| **Variable** | **Primary analysis** | **First qualifying stay** |
| --- | --- | --- |
| ICU stays | 21,342 | 19,710 |
| Unique patients | 19,874 | 19,710 |
| Early-ketamine stays | 196 | 183 |
| Black vs White | 1.63 (1.05–2.53) | 1.66 (1.04–2.63) |
| Charlson comorbidity index | 0.86 (0.79–0.93) | 0.85 (0.78–0.93) |
| SOFA score, per 1-point increase | 0.94 (0.89–0.99) | 0.94 (0.89–0.99) |
| Surgical/Trauma ICU vs Medical ICU | 2.05 (1.39–3.04) | 2.13 (1.41–3.23) |
| Cardiac ICU vs Medical ICU | 0.22 (0.11–0.42) | 0.21 (0.11–0.42) |
| P/F ratio, per 50-unit increase | 0.88 (0.82–0.96) | 0.87 (0.80–0.94) |
| MAP, per 5-mmHg increase | 1.04 (0.97–1.12) | 1.03 (0.96–1.11) |
| ≥2 vasopressors vs 0–1 | 1.07 (0.72–1.58) | 1.00 (0.66–1.52) |
| Stage 3 AKI vs Stage 0–2 | 1.67 (1.10–2.53) | 1.64 (1.04–2.59) |
| Sepsis-3: Yes vs No | 0.87 (0.61–1.24) | 0.85 (0.59–1.24) |

Values are adjusted odds ratios (95% confidence intervals), except sample counts. The first-stay complete-case analysis included 19,710 stays from 19,710 patients, including 183 early-ketamine stays. It retained the primary model covariates and natural cubic PEEP spline basis, using the same internal and boundary knots and patient-clustered robust standard errors. Selected associations are shown to match the preceding panel. PEEP was modelled nonlinearly and is not represented by a single odds ratio.

**Supplementary Table S2.** Model performance, calibration, and internal validation of the primary multivariable analysis.

| **Metric** | **Result** |
| --- | --- |
| Qualifying ICU stays in primary complete-case analysis | 21,342 |
| Unique patients in primary complete-case analysis | 19,874 |
| Early ketamine events | 196 |
| Apparent AUC | 0.832 |
| AUC 95% CI | 0.807–0.858 |
| Optimism-corrected AUC | 0.815 |
| Optimism-corrected calibration slope | 0.911 |
| Brier score | 0.0089 |
| Null Brier score | 0.0091 |
| Brier skill score | 0.025 |

AUC, area under the receiver operating characteristic curve. Optimism correction used 1,000 patient-level bootstrap resamples.

**Supplementary Table S3.** Missing data and multiple-imputation procedures

A. Missingness among primary-model covariates.

| **Variable** | **Missing, n** | **Missing, %** |
| --- | --- | --- |
| P/F ratio | 2,932 | 12.03 |
| Weight | 88 | 0.36 |
| MAP | 19 | 0.08 |
| Mean PEEP | 4 | 0.02 |
| Stage 3 AKI | 1 | <0.01 |
| Age | 0 | 0 |
| Sex | 0 | 0 |
| Race | 0 | 0 |
| First-line sedative | 0 | 0 |
| Charlson Comorbidity Index | 0 | 0 |
| SOFA score | 0 | 0 |
| ICU type | 0 | 0 |
| ≥2 vasopressors vs 0–1 | 0 | 0 |
| Sepsis-3 status | 0 | 0 |

Missing-variable counts are not mutually exclusive. Race included an unknown/not reported category and was therefore not treated as missing.

**B. Multiple-imputation procedure**

| **Characteristic** | **Result** |
| --- | --- |
| Qualifying ICU stays in study cohort | 24,363 |
| Primary complete-case ICU stays | 21,342 |
| ICU stays excluded from complete-case analysis | 3,021 |
| Number of imputations | 20 |
| Number of iterations | 10 |
| Continuous-variable method | Predictive mean matching |
| Stage 3 AKI method | Logistic regression |
| Outcome imputed | No |
| Logged MICE events | None |
| Convergence assessment | Satisfactory mixing without systematic drift |

Multiple imputation by chained equations used 20 datasets with 10 iterations. The observed outcome was included as a predictor but was not imputed. The final multivariable model was fitted separately in each completed dataset using patient-clustered robust standard errors, and estimates and variances were combined across imputations using Rubin’s rules.

**Supplementary Table S4.** Definitions and timing of variables included in the primary multivariable model.

| **Variable** | **Source** | **Timing window** | **Definition / summary rule** |
| --- | --- | --- | --- |
| Age | MIMIC-IV demographic data | Hospital admission | Age in years; modeled per 10-year increase |
| Sex | MIMIC-IV demographic data | Hospital admission | Female or Male |
| Race/ethnicity | MIMIC-IV admission data | Hospital admission | Categorized as White, Black, Asian, Hispanic, Other, or Unknown/not reported |
| Weight | mimiciv_derived.first_day_weight | First ICU day | Derived first-day body weight in kg; modeled per 10-kg increase |
| First-line continuous sedative | Study-specific inputevents query | Qualifying ICU stay | First qualifying continuous infusion of propofol, dexmedetomidine, or midazolam |
| Charlson Comorbidity Index | mimiciv_derived.charlson | Admission-level | Total Charlson Comorbidity Index |
| SOFA score | mimiciv_derived.first_day_sofa | First ICU day | Total first-day SOFA score |
| ICU type | MIMIC-IV first_careunit grouped by study-defined categories | Qualifying ICU stay | Medical, Surgical/Trauma, Cardiac, Neuro, or Mixed/Other ICU |
| P/F ratio | mimiciv_derived.first_day_bg | First ICU day | Minimum PaO₂/FiO₂ ratio; modeled per 50-unit increase |
| Mean PEEP | mimiciv_derived.ventilator_setting / study vent_summary | Entire qualifying ICU stay | Arithmetic mean of all available PEEP measurements; modeled nonlinearly using a natural cubic spline with 3 df |
| Mean arterial pressure | mimiciv_derived.first_day_vitalsign | First ICU day | Mean blood pressure (mbp_mean); modeled per 5-mmHg increase |
| Vasopressor burden | mimiciv_derived.vasoactive_agent | Entire qualifying ICU stay | Number of distinct agents among norepinephrine, epinephrine, phenylephrine, vasopressin, and dopamine; modeled as 0–1 vs ≥2 |
| Stage 3 AKI | MIMIC-IV KDIGO-derived concept | First ICU day | Stage 3 vs stages 0–2 |
| Sepsis-3 | mimiciv_derived.sepsis3 | Derived Sepsis-3 episode | Binary Sepsis-3 status |

**Supplementary Table S5.** Ketamine infusion dosing characteristics

Descriptive analysis of 220 ICU stays with identifiable continuous ketamine infusion records (97.3% of 226 early ketamine administration stays). Six additional stays had medication-push records only and were excluded from this infusion-specific analysis.

| **Characteristic** | **Result** | **Statistic / definition** |
| --- | --- | --- |
| Early-ketamine ICU stays in primary classification | 226 | Original primary cohort count |
| ICU stays with usable continuous-infusion records | 220 (97.3%) | Dosing analysis denominator |
| ICU stays excluded from dosing only | 6 (2.7%) | Isolated drug-push records; no identifiable continuous infusion |
| Initial ketamine infusion rate, mg/kg/h | 0.103 (0.100–0.300) | Median (IQR) |
| Stay-level time-weighted mean rate, mg/kg/h | 0.341 (0.192–0.603) | Median (IQR) |
| Stay-level time-weighted mean rate, mg/kg/h | 0.453 (0.405) | Mean (SD) |
| Cumulative recorded infusion duration, hours | 46.74 (18.60–148.45) | Median (IQR) |
| Cumulative recorded infusion duration, hours | 104.22 (128.48) | Mean (SD) |
| Recorded infusion segment rates, mg/kg/h | 0.006–43.103 | Minimum to maximum recorded segment |
| Cumulative recorded infusion duration, hours | 0.25–754.97 | Minimum to maximum |

Abbreviations: IQR, interquartile range; SD, standard deviation; ICU, intensive care unit. The stay-level time-weighted mean rate was calculated by weighting each qualifying recorded infusion segment’s rate by its duration. The dosing observation window extended from ketamine_start through ketamine_end without truncation at 48 hours; later qualifying segments were retained. Overlapping and contiguous segments were merged for cumulative duration only, with gaps excluded. The maximum recorded infusion-segment rate (43.103 mg/kg/h) was confirmed in the source record and corresponded to a six-minute segment. Dosing values reflect database documentation rather than independently verified administered doses; documentation errors cannot be excluded.

**Supplementary Table S6.** Primary and post hoc sensitivity analyses of early ketamine initiation.

| **Variable** | **Primary analysis** | **Excluding bolus-only stays** | **Initiation during recorded ventilation** |
| --- | --- | --- | --- |
| ICU stays | 21,342 | 21,338 | 21,289 |
| Unique patients | 19,874 | 19,871 | 19,825 |
| Early-ketamine stays | 196 | 192 | 143 |
| Age, per 10 years | 0.93 (0.82–1.04) | 0.94 (0.84–1.06) | 0.90 (0.78–1.02) |
| Male vs female | 0.95 (0.71–1.29) | 0.94 (0.69–1.28) | 1.00 (0.70–1.43) |
| Black vs White | 1.63 (1.05–2.53) | 1.68 (1.08–2.60) | 1.46 (0.87–2.45) |
| Asian vs White | 1.15 (0.47–2.84) | 1.18 (0.48–2.91) | 1.29 (0.46–3.62) |
| Hispanic vs White | 1.05 (0.55–2.00) | 0.96 (0.49–1.88) | 1.32 (0.67–2.60) |
| Other race vs White | 0.81 (0.39–1.69) | 0.81 (0.39–1.70) | 0.86 (0.36–2.02) |
| Unknown/not reported vs White | 0.74 (0.50–1.09) | 0.72 (0.48–1.07) | 0.73 (0.46–1.14) |
| Weight, per 10 kg | 0.99 (0.95–1.04) | 0.99 (0.95–1.04) | 1.00 (0.95–1.05) |
| Dexmedetomidine vs propofol | 0.82 (0.20–3.34) | 0.86 (0.21–3.50) | 1.02 (0.25–4.17) |
| Midazolam vs propofol | 0.73 (0.50–1.07) | 0.76 (0.51–1.11) | 0.71 (0.46–1.10) |
| Charlson index, per point | 0.86 (0.79–0.93) | 0.84 (0.77–0.91) | 0.89 (0.81–0.97) |
| SOFA score, per point | 0.94 (0.89–0.99) | 0.94 (0.89–0.99) | 0.96 (0.91–1.02) |
| Surgical/Trauma vs Medical ICU | 2.05 (1.39–3.04) | 2.12 (1.42–3.16) | 1.49 (0.97–2.31) |
| Cardiac vs Medical ICU | 0.22 (0.11–0.42) | 0.22 (0.11–0.42) | 0.18 (0.09–0.39) |
| Neuro vs Medical ICU | 1.08 (0.41–2.86) | 1.13 (0.43–3.02) | 1.33 (0.49–3.59) |
| Mixed/Other vs Medical ICU | 1.10 (0.68–1.76) | 1.14 (0.71–1.85) | 1.04 (0.62–1.75) |
| P/F ratio, per 50 units | 0.88 (0.82–0.96) | 0.89 (0.82–0.97) | 0.93 (0.84–1.02) |
| MAP, per 5 mmHg | 1.04 (0.97–1.12) | 1.05 (0.98–1.13) | 1.06 (0.97–1.15) |
| ≥2 vasopressors vs 0–1 | 1.07 (0.72–1.58) | 1.02 (0.68–1.52) | 1.02 (0.65–1.61) |
| Stage 3 AKI vs stages 0–2 | 1.67 (1.10–2.53) | 1.62 (1.05–2.48) | 1.72 (1.07–2.75) |
| Sepsis vs no sepsis | 0.87 (0.61–1.24) | 0.88 (0.61–1.26) | 1.21 (0.75–1.95) |

Values are adjusted odds ratios (95% confidence intervals), except sample counts. Both post hoc analyses were conducted separately from the primary complete-case analysis and retained the original covariates, PEEP spline basis, patient-clustered robust standard errors and 21,146 no-early-ketamine comparison stays. Four of six bolus-only stays and 53 of 61 starts outside recorded invasive ventilation were present in the primary complete-case model and excluded from their respective sensitivity analyses. The other two and eight stays, respectively, were already absent because of missing covariates. Excluded stays were removed, not reclassified as controls; the two restrictions were not combined.

**Supplementary Table S7.** Timing of early ketamine administration relative to recorded invasive ventilation

Recorded ketamine initiation for all 226 early classified ICU stays was compared with the documented invasive ventilation intervals within the same stay.

| **Start time classification** | **ICU stays** | **Percent of 226** |
| --- | --- | --- |
| During a recorded invasive ventilation interval | 165 | 73.0% |
| After the last recorded invasive ventilation interval | 28 | 12.4% |
| Before the first recorded invasive ventilation interval | 23 | 10.2% |
| Between recorded invasive ventilation intervals | 10 | 4.4% |
| Total | 226 | 100.0% |

Among the 28 starts after the last recorded interval, the gap had a median of 8.03 hours (range 0.63–33.53 hours); 26 occurred more than one hour after the interval ended. These classifications reflect recorded event times and do not confirm extubation or the clinical indication for treatment. Initiation during ventilation was defined as occurring at or after an interval start and before its end. Percentages use all 226 early-ketamine stays, rather than the complete-case regression sample.

**Supplementary Figure S1. Adjusted nonlinear association between mean PEEP and early ketamine initiation**. The solid line represents adjusted odds ratios estimated from the primary multivariable logistic regression model using a natural cubic spline with three degrees of freedom, with patient-clustered robust standard errors; the shaded area represents the 95% confidence interval. Odds ratios are referenced to the median mean PEEP of 5.15 cm H₂O. Predictions are displayed over the 1st to 99th percentile of observed mean PEEP values. Mean PEEP was associated with early ketamine initiation overall (P < 0.001), with evidence of nonlinearity (P = 0.005).
