# Supplementary material for: Clinical Factors Associated With Early Ketamine Initiation Among Mechanically Ventilated Adults: A Retrospective MIMIC‐IV Cohort Study
Source: Anesthesiol Res Pract. 2026 Nov 4;2026:3312071. doi: 10.1155/anrp/3312071 (PMC13732811; doi:10.1155/anrp/3312071)
Supplement: Supplementary file 2 [file ANRP-2026-3312071-s002.docx]

**STROBE Statement — Completed Checklist for Cohort Studies**

**Manuscript: Clinical factors associated with early ketamine initiation during mechanical ventilation: a retrospective MIMIC-IV cohort study**

Authors: Mary Ivasyutyn, Gaurav Prabhu, and Saajan Bhakta

*Checklist based on the STROBE cohort-study reporting guideline. Page references correspond to the current manuscript draft.*

| **STROBE section/topic** | **Item** | **Reporting requirement** | **Where reported in manuscript** |
| --- | --- | --- | --- |
| Title and abstract | 1a | Identify the observational cohort design in the title or abstract. | p. 1 — title states “retrospective MIMIC-IV cohort study”. |
| Title and abstract | 1b | Provide an informative, balanced summary of methods and findings. | p. 2 — structured Summary (Introduction, Methods, Results, Discussion). |
| Introduction — Background/rationale | 2 | Explain the scientific background and rationale. | pp. 3–4 — ICU sedation context, ketamine evidence, and knowledge gap. |
| Introduction — Objectives | 3 | State the study objective and any prespecified hypotheses. | p. 4 — objective is to examine clinical correlates of early ketamine initiation. |
| Methods — Study design | 4 | Present key elements of the study design early. | p. 4 — retrospective observational cohort study using MIMIC-IV v3.1. |
| Methods — Setting | 5 | Describe setting, location, and relevant study/data-collection dates. | p. 4 — MIMIC-IV v3.1, Beth Israel Deaconess Medical Center, Boston; data span 2008–2022. |
| Methods — Participants | 6a | Describe eligibility criteria and how participants/stays were selected. | pp. 4–5 — adults ≥18 years; invasive mechanical ventilation; qualifying continuous first-line sedation; ICU stay as unit of analysis. |
| Methods — Participants | 6b | For matched studies, report matching criteria and numbers. | Not applicable — this was not a matched cohort study. |
| Methods — Variables | 7 | Clearly define outcome and model covariates; give diagnostic criteria where relevant. | p. 5 and Supplementary Table S4 — early ketamine definition, covariates, KDIGO stage 3 AKI, Sepsis-3, and timing definitions. |
| Methods — Data sources/measurement | 8 | Describe data sources and how variables were measured/derived. | pp. 5–6 and Supplementary Table S4 — MIMIC-IV-derived concepts, official MIT-LCP SQL concepts, custom PostgreSQL queries, variable-specific windows. |
| Methods — Bias | 9 | Describe efforts to address potential sources of bias. | pp. 5–7 and p. 10 — episode definition, patient-level clustering, complete-case analysis, multiple imputation, Firth regression, strict-timing and first-stay sensitivity analyses; timing overlap and residual bias discussed as limitations. |
| Methods — Study size | 10 | Explain how the study size was determined. | pp. 6–7 — no a priori power calculation; all eligible MIMIC-IV ICU stays were included. |
| Methods — Quantitative variables | 11 | Explain how quantitative variables were handled and any groupings/scaling. | pp. 6–7 and Table 2 (pp. 20–21) — continuous scaling specified; P/F retained linear; mean PEEP modelled with a 3-df spline; categorical reference groups defined. |
| Methods — Statistical methods | 12a | Describe statistical methods, including those used to control for confounding. | pp. 6–7 — descriptive tests, multivariable logistic regression, patient-clustered robust standard errors, prespecified covariates, spline modelling. |
| Methods — Statistical methods | 12b | Describe subgroup or interaction analyses, if performed. | Not applicable — no prespecified subgroup or interaction analyses were performed. |
| Methods — Statistical methods | 12c | Explain how missing data were handled. | pp. 6–7 and Supplementary Table S3 — complete-case primary analysis; 20-dataset MICE sensitivity analysis with Rubin pooling and clustered robust variance. |
| Methods — Statistical methods | 12d | Explain handling of loss to follow-up, if applicable. | Not applicable — retrospective ICU episode analysis with a fixed 48-hour outcome window; no longitudinal follow-up cohort. |
| Methods — Statistical methods | 12e | Describe sensitivity analyses. | pp. 6–8 and Supplementary Table S1 — no-P/F, multiple imputation, Firth, strict timing, and first-stay analyses. |
| Results — Participants | 13a | Report numbers at key stages of inclusion and analysis. | p. 7 and Figure 1 — 24,363 qualifying stays/22,396 patients; 3,021 excluded from complete-case analysis; 21,342 complete cases. |
| Results — Participants | 13b | Give reasons for exclusions/non-participation. | Figure 1 and pp. 7–8 — primary complete-case exclusions attributed to missing model covariates; stricter timing exclusions reported in sensitivity analysis. |
| Results — Participants | 13c | Consider a participant flow diagram. | Figure 1 — study cohort and primary analytic sample flowchart. |
| Results — Descriptive data | 14a | Report participant characteristics and relevant clinical variables. | Table 1, pp. 16–18. |
| Results — Descriptive data | 14b | Report missing data for variables of interest. | Table 1 footnote (p. 18) and Supplementary Table S3. |
| Results — Descriptive data | 14c | Summarise follow-up time, if relevant. | Not applicable — outcome was early ketamine initiation within a fixed 48-hour window. |
| Results — Outcome data | 15 | Report the number of outcome events. | pp. 2 and 7 — 226 early-ketamine events (0.9%); 196 events in the primary complete-case analysis. |
| Results — Main results | 16a | Report unadjusted and adjusted estimates with precision and clarify adjustment variables. | Table 1 (unadjusted comparisons); pp. 7–8 and Table 2 (adjusted ORs with 95% CIs); covariate selection described on p. 5. |
| Results — Main results | 16b | Report category boundaries for categorised continuous variables. | Table 2 footnote, pp. 20–21 — reference categories and grouped variables (e.g., ≥2 vs 0–1 vasopressors; AKI stage 3 vs 0–2). |
| Results — Main results | 16c | Where useful, translate relative estimates into absolute risk. | Not applicable — study objective was association/practice-pattern characterization; overall event rate (0.9%) is reported. |
| Results — Other analyses | 17 | Report sensitivity, subgroup, interaction, or other analyses. | pp. 7–8; Supplementary Tables S1–S3 and Supplementary Figure S1. |
| Discussion — Key results | 18 | Summarise key findings in relation to the objective. | pp. 8–10 — opening Discussion paragraph and subsequent interpretation. |
| Discussion — Limitations | 19 | Discuss limitations, potential bias, and imprecision. | p. 10 — retrospective design, nonuniform timing, small ketamine-event group, unmeasured longitudinal treatment changes, and single-centre database. |
| Discussion — Interpretation | 20 | Provide a cautious interpretation considering limitations and existing evidence. | pp. 8–10 — findings framed as clinical correlates/practice patterns rather than causal effects; compared with prior literature. |
| Discussion — Generalisability | 21 | Discuss external validity/generalisation. | p. 10 — single academic health system limitation and need for validation in other populations/settings. |
| Other information — Funding | 22 | Report funding source and funder role. | p. 11 — no specific grant funding. |

**Additional transparency statements (not separate STROBE checklist items):** Ethics/data governance, competing interests, data availability, and code availability are reported on p. 11.

**Pre-submission note:** *Update page references if pagination changes after final journal-style edits.*
